# Identifying the characteristics of patients with stroke who have difficulty benefiting from gait training with the hybrid assistive limb: a retrospective cohort study

**DOI:** 10.3389/fnbot.2024.1336812

**Published:** 2024-02-08

**Authors:** Shingo Taki, Takeshi Imura, Tsubasa Mitsutake, Yuji Iwamoto, Ryo Tanaka, Naoki Imada, Hayato Araki, Osamu Araki

**Affiliations:** ^1^Department of Rehabilitation, Araki Neurosurgical Hospital, Hiroshima, Japan; ^2^Graduate School of Humanities and Social Sciences, Hiroshima University, Higashihiroshima, Japan; ^3^Department of Rehabilitation, Faculty of Health Sciences, Hiroshima Cosmopolitan University, Hiroshima, Japan; ^4^Department of Physical Therapy, Fukuoka International University of Health and Welfare, Fukuoka, Japan; ^5^Department of Neurosurgery, Araki Neurosurgical Hospital, Hiroshima, Japan

**Keywords:** stroke, hybrid assistive limb, walking independence, functional ambulation category, logistic regression analysis

## Abstract

Robot-assisted gait training is effective for walking independence in stroke rehabilitation, the hybrid assistive limb (HAL) is an example. However, gait training with HAL may not be effective for everyone, and it is not clear who is not expected to benefit. Therefore, we aimed to identify the characteristics of stroke patients who have difficulty gaining benefits from gait training with HAL. We conducted a single-institutional retrospective cohort study. The participants were 82 stroke patients who had received gait training with HAL during hospitalization. The dependent variable was the functional ambulation category (FAC) that a measure of gait independence in stroke patients, and five independent [age, National Institutes of Health Stroke Scale, Brunnstrom recovery stage (BRS), days from stroke onset, and functional independence measure total score (cognitive items)] variables were selected from previous studies and analyzed by logistic regression analysis. We evaluated the validity of logistic regression analysis by using several indicators, such as the area under the curve (AUC), and a confusion matrix. Age, days from stroke onset to HAL initiation, and BRS were identified as factors that significantly influenced walking independence through gait training with HAL. The AUC was 0.86. Furthermore, after building a confusion matrix, the calculated binary accuracy, sensitivity (recall), and specificity were 0.80, 0.80, and 0.81, respectively, indicated high accuracy. Our findings confirmed that older age, greater degree of paralysis, and delayed initiation of HAL-assisted training after stroke onset were associated with increased likelihood of walking dependence upon hospital discharge.

## 1 Introduction

Stroke affects 15 million people worldwide annually (World Health Organization, 2015) and causes a variety of after-effects. Approximately 64% of patients with stroke have significant disabilities (e.g., walking disability) due to sensory and motor impairments (Patel et al., 2006). Gaining walking independence has been reported to be critical for the patient's quality of life after a stroke (Chang et al., 2016). Thus, gaining walking independence is one of the biggest objectives for patients recovering from stroke.

To regain their walking independence, patients with stroke are recommended to undergo repetitive gait training as a part of their rehabilitation program (Peurala et al., 2014). A systematic review of the current guidelines (Calabro et al., 2021) indicates that robotic-assisted gait training supports repetitive walking exercises, increases neuroplasticity, and improves motor skills. The hybrid assistive limb (HAL) is an example of a gait training device which in combination with the conventional gait rehabilitation program improves walking independence in patients with stroke (Taki et al., 2020). The prognosis of patients with stroke in terms of walking ability may be improved further through robotic rehabilitation (Baronchelli et al., 2021; Calafiore et al., 2022). On the other hand, it is far from established and recommended when, how and for whom to use a particular device (Morone et al., 2017). A previous study suggested that patients with stroke who are pain-free, have good communication, and visual–spatial function are more suitable for walking training with HAL (Chihara et al., 2016). Another study indicated that HAL is more effective for patients with stroke who have less lower limb paralysis, such as Brunnstrom recovery stage (BRS) III or more in acute rehabilitation after stroke (Fukuda et al., 2015). In facts, during clinical practice, as clear characteristics of patients are unknown, it is difficult to identify which patients should receive gait training with HAL; some patients even fail to exhibit sufficient therapeutic response for robot-assisted gait training. A systematic review of current guidelines on robotic-assisted gait rehabilitation after stroke states that future guidelines should focus on identifying the characteristics of patients who may better benefit from a specific robotic device (Morone et al., 2017). In addition, a systematic review of effects of gait training with HAL reported no strong evidence supporting the effectiveness of HAL in improving the walking ability owing to differing patient characteristics (Taki et al., 2022). Therefore, identifying the characteristics of patients who have difficulty gaining benefits from gait training with HAL is crucial, as it provides criteria for clinical decision-making, including the arrangement of individual rehabilitation programs, early discharge planning and guideline development.

The age (Carod-Artal and Egido, 2009), National Institutes of Health Stroke Scale (NIHSS) (Koenig et al., 2008), the severity of lower limb paralysis (Jørgensen et al., 1995), the number of days from stroke onset (Kim et al., 2016), and functional independence measure (FIM) total score (cognitive items) (Ozdemir et al., 2001) were reported that influence walking independence in patients with stroke. These factors may be related to the effect of gait training with HAL on walking independence. Therefore, we aimed to identify the characteristics of patients with stroke who have difficulty gaining benefits from gait training with HAL.

## 2 Materials and methods

### 2.1 Study design

We conducted a single-institutional retrospective cohort study with the approval of the Ethical Review Committee of the Araki Neurosurgical Hospital (2021-10). Including the publication of details comprehensive written informed consent was obtained from all participants in this study. If the participants were minors, informed consent was to be provided to their parents or guardians, but minors were not among the participants in this study. Specific informed consent regarding the implementation of present observational study was waived because of the retrospective nature of this study. All participants were provided opportunities to withdraw consent. This study was conducted in accordance with the Declaration of Helsinki.

### 2.2 Setting

The participants were patients with stroke with hemiplegia who were admitted to and discharged from our facility from April 2017 to July 2021. With the approval of the Ethical Review Committee, we started accessing and collecting data on participations from 13th August 2021. In the process, information that can immediately identify a specific individual (name, address, medical record number, etc.) was not used, but a code or number was assigned to the research subject and a pseudonym was processed to create a collation table. The collation table was kept by the principal investigator and was never taken outside the institution. Furthermore, only the principal investigators had access to them.

### 2.3 Participants

Participants were selected based on the following inclusion criteria: patients who (i) had received a diagnosis of stroke from a physician based on CT and MRI imaging findings; (ii) had a stroke within the last 6 months; (iii) had performed gait training with HAL Lower Limb type; (iv) had been evaluated as BRS (Imura et al., 2015) lower limb II–V; and (v) were able to understand simple verbal commands. Patients were excluded if; (i) they had been evaluated by a physician as likely to be unable to carry out rehabilitation have difficulty in carrying out rehabilitation; (ii) they had acquired cerebrovascular disorders or orthopedic diseases during hospitalization; (iii) they had skin diseases that prevented the attachment of electrodes; (iv) they did not receive consent to be treated with HAL; (v) they had not received consistent rehabilitation at a single facility since the onset of illness; and (vi) they had a walking independence score of 3 or less before their stroke on the functional ambulation category (FAC) (Holden et al., 1986). Additionally, we excluded patients who dropped out during gait training with HAL from the analysis.

### 2.4 Rehabilitation program

Under the Japanese medical insurance system, a medical doctor prescribes a rehabilitation program to patients with stroke, and rehabilitation therapists such as physiotherapists, occupational therapists, and speech-language-hearing therapists arrange individual rehabilitative exercise programs for up to 3 h per day (Imura et al., 2018; Iwamoto et al., 2019), 7 days per week. The rehabilitation program is based on conventional programs for patients with stroke. It includes passive or active mobilization, sitting, standing, gait training with knee-ankle-foot orthosis or ankle-foot orthosis, climbing stairs, activities of daily living (ADL) training, speech training, swallowing training, cognitive training, and coaching for family or caregivers, depending on the patient's symptoms and goals. In addition to the above training, the patients in this study underwent gait training with HAL for at least 40 min per day (including the rest time), up to three times per week. On the days when HAL was performed, gait training with orthotics was canceled. HAL for wellbeing (double-leg model, lower limb type; CYBERDYNE Inc., Tsukuba, Japan) was used for gait training. The following are characteristics of HAL: (i) HAL senses bioelectrical signals at the skin surface; these signals are intended for the peripheral nerves from the brain. (ii) Based on these signals, HAL determines the patient's intended limb movements and responds by inducing and supporting normal movement patterns. (iii) To rehabilitate patients to normal gait movement patterns, the patients provide sensory feedback at appropriate times by allowing them to express movements at certain times from an early stage in the treatment (Kawamoto et al., 2013; Saita et al., 2018). HAL offers three control systems: cybernic voluntary control (CVC), cybernic impedance control (CIC), and cybernic autonomous control (CAC). In CVC mode, assistance for patients' motion is triggered by bioelectrical signals intended for the peripheral nerves. CIC mode enables the production of smooth joint movements by reducing the HAL suit's joint resistance. In CAC mode, the patient's movement is autonomously supported by determining the standing or swing phase through force-pressure sensors in the shoes. During gait training with HAL, physical therapists selected HAL control systems based on the patient's condition. In the present study, the CVC mode was predominantly used for both the paralyzed and nonparalyzed sides.

### 2.5 Variables

#### 2.5.1 Outcome

We used FAC to assess functional outcomes and collected data from medical records and the hospital's database at the time of hospitalization and upon hospital discharge. FAC measured the degree of walking on a 6-point scale, where 0 represents “non-ambulatory” and 5 represents “ambulatory independent” (Holden et al., 1986).

#### 2.5.2 Potential predictors

Based on previous studies (Jørgensen et al., 1995; Ozdemir et al., 2001; Koenig et al., 2008; Carod-Artal and Egido, 2009; Kim et al., 2016), age, NIHSS (Goldstein et al., 1989), BRS of the lower limb, the number of days from stroke onset to HAL initiation and FIM (Granger et al., 1993) total score (cognitive items) was selected as the potential predictor, collected from medical records, and the hospital's database at the time of hospitalization and upon hospital discharge. BRS and NIHSS were used to assess functional outcomes; FIM measured ADL ability outcome. BRS evaluates the statuses of the upper limbs, fingers, and lower limbs on a 6-point scale, with stage I representing flaccid paralysis and stage VI representing possible separation movement. NIHSS uses 13 items to assess the level of consciousness, language, neglect, visual-field loss, extraocular movements, motor strength, ataxia, dysarthria, and sensory loss. FIM uses 13 items to assess motor function and five items to assess cognition. All the clinical outcomes were evaluated by the attending rehabilitation therapist.

#### 2.5.3 Potential confounders

The following information was collected from medical records and the hospital's database as potential confounders: age, sex, stroke type, paresis side, the number of days from stroke onset to admission, and the number of days from stroke onset to rehabilitation prescription. In addition, we collected HAL-related data, including the number of days from stroke onset to HAL initiation, the total number of times that HAL was used, the frequency of HAL use per week, and the type of lower limb orthosis at HAL initiation.

### 2.6 Bias

Many variables can affect gait independence in patients with stroke. To avoid omitted variable bias, we selected independent variables to include in logistic regression analysis based on previous studies.

### 2.7 Statistical method

All statistical analyses were performed in JMP Pro version 16 (JMP Japan, Roppongi, Tokyo, Japan). *p*-values of < 0.05 were considered statistically significant.

We classified patients who were walking independently at discharge into the independent group (IG, FAC score of 4 or more points) and those who were walking dependently into the dependent group (DG, FAC score of < 4 points) by using the FAC scores at hospital discharge. We then compared and analyzed to examine trends in the baseline characteristics of patients between the two groups. Mann–Whitney *U*-tests and chi-squared tests were used for comparisons, with *p* < 0.05 indicating statistical significance.

We used logistic regression analysis for identifying the characteristics of patients with stroke who have difficulty gaining benefits from gait training with HAL. In this study, patients who achieved walking independence (FAC score of ≥4 points) were designated as “independent” while those with FAC scores < 4 points were designated as “dependent” (Kollen et al., 2005).

The independent variables were selected age, NIHSS on admission, BRS of the lower limb on admission, the number of days from stroke onset to HAL initiation, and FIM total score (cognitive items) at admission. Moreover, to identify which variables in the acute phase were predictors of walking independence (FAC) at hospital discharge, logistic regression analysis was performed after setting FAC (Binarization of dependent (FAC score of < 4 points) and independent (FAC score of ≥4 points) as the dependent variable (Kollen et al., 2005), and calculating the *p*-value, the odds ratio (OR), and the 95% confidence intervals (95% CI). Predictors for walking independence were selected with stepwise forward selection, using the Akaike information criterion (AIC Akaike, 1973) as a stopping rule. Finally, the number of independent variables was set to ≤ 4. In addition, rank-order correlation (rho) was used to evaluate multicollinearity between independent variables. If a strong correlation (rho ≥ 0.7) was detected, then the independent variables were not used in the same logistic regression analysis. Validity of logistic regression analysis was evaluated using several indicators, such as the Nagelkerke *R*^2^ test, receiver operating characteristics curve (ROC), and confusion matrix (Kubben et al., 2019). The Nagelkerke *R*^2^ test yields values between 0 and 1, where closer proximity to 1 indicates a better goodness of fit. The area under the curve (AUC) was estimated from the ROC and validated internally using bootstrap resampling (Zemek et al., 2016). Furthermore, a confusion matrix was used to evaluate the binary accuracy, sensitivity (recall), and specificity. In addition, we performed ROC analysis using FAC and applied the Youden Index to determine the cutoff values for the selected factors. The Youden Index, a metric combining sensitivity and specificity, serves as a widely employed measure of overall diagnostic effectiveness (Youden, 1950). The maximum value of the index can be used as a criterion for selecting the optimal cutoff point (Powers, 2011).

### 2.8 Sample size

The target number of patients in each group was set at 50 because the number of independent variables for the multiple logistic regression was defined as the number of events in the dependent variables divided by the number of independent variables ≥10 (Peduzzi et al., 1996).

## 3 Results

### 3.1 Participant flow

**Figure 1:**
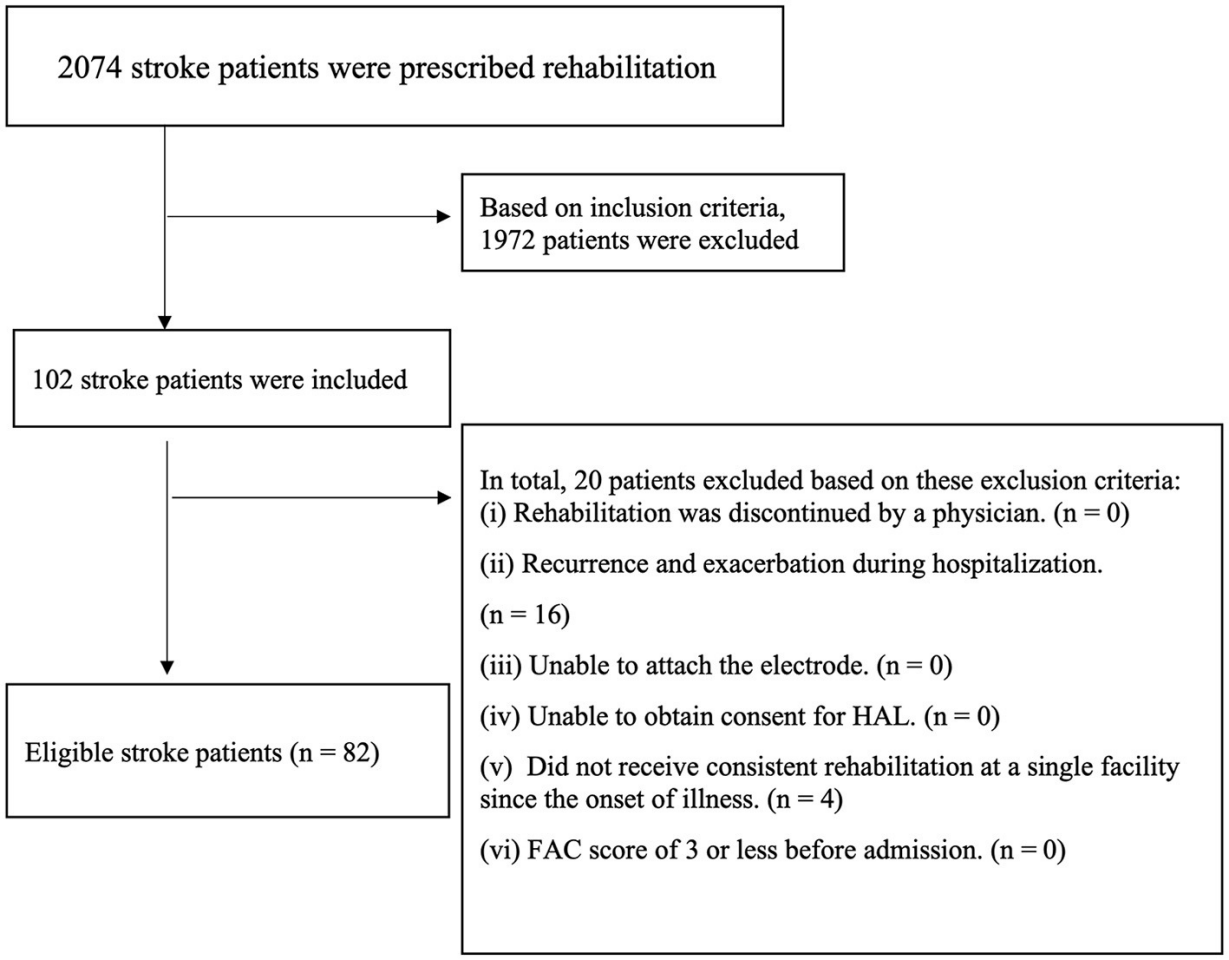
Study flowchart. Logistic regression analyses were performed for 82 stroke patients.

The participants consisted of 2,074 patients with stroke having hemiplegia who were hospitalized and discharged to our facility from April 2017 to July 2021. We analyzed 82 participants after screening the patients with the inclusion and exclusion criteria (Figure 1). No patients were excluded during gait training with HAL, and the mean and median number of hospitalization days for patients was 112.9 days and 112.5 days, respectively.

### 3.2 Comparison between groups at baseline

Of 82 participants, 40 and 42 patients were classified as IG and DG, respectively. The between-group comparisons of the independent variable at the time of hospitalization are summarized in Table 1. Mann–Whitney *U*-tests showed that mean age, the median NIHSS at admission, the median BRS at admission, the mean FIM score (total score of cognitive items on an ADL scale) at admission, and the number of days from stroke onset to initiation of gait training were significantly different (*p* < 0.05) between groups. Chi-squared tests showed that the number of each sex, stroke type, and side of paresis were not significantly different between groups.

**Table 1:** Comparison of baseline characteristics of patients who gait training in HAL between groups.

	**DG (*n* = 42)**	**IG (*n* = 40)**	***p-*value**
Age, years old (SD)	71.3 (11.8)	63.5 (10.3)	0.00^†^
**Sex, no**			0.48^‡^
Male	23	25	
Female	19	15	
**Stroke type, no**			0.52^‡^
Hemorrhagic	25	21	
Ischemic	17	19	
**Side of paresis, no**			0.84^‡^
Right	23	21	
Left	19	19	
The number of days from stroke onset to admission, days (SD)	1.4 (3.1)	1.2(4.2)	0.74^†^
The number of days from stroke onset to rehabilitation prescription, days (SD)	1.6 (2.5)	1.9 (4.3)	0.40^†^
The number of days from stroke onset to initiation of gait training, days (SD)	9.0 (5.6)	6.8 (5.7)	0.01^†^
NIHSS at admission, score (IQR)	13 (8–17)	8 (5–12)	0.00^†^
BRS at admission (IQR)	2 (2–3)	3 (2–4)	0.00^†^
FIM (total score of cognitive items) at admission, score (SD)	14.9 (7.6)	19.8 (7.9)	0.00^†^
**FAC at admission, no**			
FAC 0	2	0	
FAC 1	9	0	
FAC 2	15	0	
FAC 3	16	0	
FAC 4	0	21	
FAC 5	0	19	

DG, dependent group; IG, independent group; IQR, interquartile range; SD, standard deviations; BRS, Brunnstrom recovery stage (lower limb); NIHSS, National Institutes of Health Stroke Scale; FIM, functional independence measure; FAC, functional ambulation category.

^†^Mann–Whitney U-test.

^‡^Chi-squared test.

The between-group comparisons of orthosis usage and HAL data characteristics are summarized in Table 2. Results of Mann–Whitney *U*-tests show the number of days from stroke onset to initiation of HAL [IG: 21.7 (standard deviations (SD) 12.1), DG: 38 (SD 21.2)] differ significantly between groups (*p* < 0.05), while Mann–Whitney *U*-tests and chi-squared tests showed that the total number of times HAL was used, frequency of HAL usage within a week, and types of lower limb orthotics used at the start of HAL did not differ significantly between groups.

**Table 2:** Comparison of HAL data characteristics of patients who gait training with HAL between groups.

	**DG (*n* = 42)**	**IG (*n* =40)**	***p-*value**
The number of days from stroke onset to initiation of HAL, days (SD)	38.0 (21.2)	21.7 (12.1)	0.00^†^
Total number of HAL use, times (SD)	8.5 (5.5)	8.5 (6.2)	0.84^†^
Frequency of HAL use in a week, times (SD)	2.7 (1.5)	3.4 (2.4)	0.28^†^
**HAL control systems, no**			
**Paralyzed side**			
CVC	42	40	
CAC	0	0	
CIC	0	0	
**Non-paralyzed side**			
CVC	42	40	
CAC	0	0	
CIC	0	0	
**Types of lower limb orthosis at initiation of HAL, no**			0.90^‡^
KAFO	27	24	
AFO	5	5	
No use	10	11	

HAL, hybrid assistive limb; KAFO, knee–ankle–foot-orthosis; AFO, ankle–foot-orthosis; CVC, cybernic voluntary control; CAC, cybernic autonomous control; CIC, cybernic impedance control.

^†^Mann–Whitney U-test.

^‡^Chi-squared test.

### 3.3 Logistic regression analysis

The stepwise forward selection was used to select variables for logistic regression analysis, and age, BRS, and days from stroke were selected as the independent variables. Logistic regression analysis shows that age (OR: 1.08, 95% CI: 1.03–1.14, *p* < 0.00), BRS (OR: 0.49, 95% CI: 0.30–0.80, *p* < 0.00), and days from stroke onset to HAL initiation (OR: 1.06, 95% CI: 1.02–1.10, *p* < 0.00) were significantly associated with FAC at discharge (Table 3). ROC analysis revealed that cutoff values for age, BRS, and days from stroke onset to HAL initiation were 71 years (AUC = 0.72; *p* < 0.00), stage II (AUC = 0.69; *p* < 0.00), and 33 days (AUC = 0.76; *p* < 0.00). The Nagelkerke *R*^2^ test was used to evaluate the validity of logistic regression analysis, and our calculations generated a value of 0.30. This result indicates that the goodness of fit was not notably high. However, an AUC value of 0.86 was calculated from the ROC curve, and bootstrapping analysis (i.e., resampling the model 1,000 times) revealed a corrected AUC value of 0.86. Furthermore, upon constructing a confusion matrix, the calculated binary accuracy, sensitivity (recall), and specificity were 0.80, 0.80, and 0.81, respectively, indicating high accuracy.

**Table 3:** Associations between walking independence (functional ambulation category) at hospital discharge and each variable in the acute phase.

**Independent variables**	**OR**	**95% CI**	***p-*value**
Age	1.08	1.03–1.14	0.00
BRS at admission	0.49	0.30–0.80	0.00
The number of days from stroke onset to initiation of HAL	1.06	1.02–1.10	0.00

BRS, Brunnstrom recovery stage (lower limb); OR, odds ratio; 95% CI, 95% confidence interval; HAL, hybrid assistive limb.

## 4 Discussion

The present study aimed to identify the characteristics of patients with stroke who have difficulty benefiting from gait training with HAL. The study included 82 patients with stroke who performed gait training with HAL. After selecting predictors for walking independence with stepwise forward selection, logistic regression analysis showed that age, BRS, and days from stroke onset to HAL initiation were significantly associated with FAC at discharge.

To the best of our knowledge, this is the first report to identify the characteristics of patients with stroke who experience difficulty benefiting from gait training with HAL. Previously, several factors [such as age, NIHSS, the severity of lower limb paralysis, number of days, and FIM total score (cognitive items)] have been reported to influence walking independence in patients with stroke, but the influence on walking independence using HAL was not known. In our study, we identified that the characteristics of patients with stroke who used HAL, such as age, days from stroke onset to HAL initiation, and the severity of lower limb paralysis, were the most significant factors influencing walking independence in these patients. Furthermore, we revealed the cutoff values of these characteristics and assessed the accuracy of the model. We believe that these results are novel.

Our findings fill a gap in our knowledge about the patient indications for HAL. The characteristics that influence walking independence in patients with stroke have been investigated in many studies, and patient age and the days from stroke onset to gait training are recognized as factors that influence the prognosis of patients (Carod-Artal and Egido, 2009; Imura et al., 2018). In the background of the older adults, there is degeneration of the neuromuscular system associated with aging (Jang and Remmen, 2011). Additionally, the prevalence of sarcopenia is higher in the elderly (Morley, 2012*)*. It has been reported that older patients with stroke experience poorer outcomes (Sprigg et al., 2013) and age is considered a factor affecting the walking independence of patients with stroke (Carod-Artal and Egido, 2009). The acute phase following a stroke is a period of flexible plastic changes in the brain. Notably, the most pronounced functional recovery occurs within the first 30 days after stroke onset, regardless of initial stroke severity (Duncan et al., 1992). Furthermore, a study advocating the early initiation of gait training with HAL (Degami et al., 2023) suggested that starting gait training using HAL in the acute period may offer numerous benefits in enhancing the motor function of the affected lower limb. The identified characteristics of patients with stroke support these previous findings, suggesting that these factors play consistent roles in patient prognosis, even across different types of rehabilitation programs. We also identified the severity of lower limb paralysis at admission as another factor that influences the effects of gait training with HAL in patients with stroke. A previous study focused on the effects of the severity of lower limb paralysis on human intent-controlled robot-assisted motor rehabilitation (Li et al., 2018) suggested that continuous electromyography-based control may reinforce pathological behavior rather than promote the recovery of normal movement patterns in patients with stroke. Because many patients with stroke have paralyzed muscles that contract abnormally. Therefore, HAL could not accurately sense the intentions of a patient with severe paralysis and may instead reinforce pathological movements, indicating that the need for HAL intervention should be based partly on the severity of paralysis.

Our study provides important insights for clinicians who want to use HAL effectively. A previous study suggested that gait training with HAL improves walking speed and distance and leads to walking independence regardless of age, sex, and severity of paralysis of the lower limbs (Wall et al., 2015). However, our analysis confirms that the probability of walking independence after hospital discharge in patients with stroke undergoing gait training with HAL is affected by age, the degree of paralysis, and the time from stroke onset to the start of HAL training. Our analysis helps to resolve the controversy concerning the influence of these factors on gait training with HAL. This information provides criteria for clinical decision-making, including the strategy for individual rehabilitation programs and early discharge planning. Furthermore, the supply of costly devices, including HAL, is limited; therefore, standardized criteria for determining the need for gait training with HAL would help promote its use.

Our study has several limitations. First, our groups did not reach the target number of 50 patients. However, considering that only three independent variables were included in the characteristics of patients with stroke that were significantly associated with FAC at discharge and the number of participants in each group exceeded 30 patients, it is likely that we provide reliable analysis results. Second, our logistic regression analysis relied on a limited number of variables because our sample size was about only 40 per group. Therefore, the characteristics of the output of patients with stroke obtained using logistic regression should be interpreted with caution, as other confounding factors cannot be ruled out. Nevertheless, as shown in Tables 1, 2, analysis of the patient data used in this study shows that, other than the independent variables incorporated in the characteristics of patients with stroke were significantly associated with FAC at discharge, no other variable differed significantly between the two groups of patients. Therefore, the characteristics of patients with stroke that we identified may provide reliable analysis results despite the small number of variables. Third, our results should not be generalized to other populations, because our study was conducted at a single institution, and each facility has a different environment and rehabilitation system. Further, to clarify the relationship between the characteristics of patients identified in this study and walking independence, the results need to be validated through a multicenter study with a larger sample size.

## 5 Conclusions

We identified the characteristics of patients with stroke who have difficulty gaining benefits from gait training with HAL among patients with stroke. Our findings confirm that older age, greater degree of paralysis, and delayed initiation of HAL-assisted training after stroke onset were associated with increased likelihood of walking dependence upon hospital discharge.

## Data availability statement

The original contributions presented in the study are included in the article/supplementary material, further inquiries can be directed to the corresponding author.

## Ethics statement

The studies involving humans were approved by the Ethical Review Committee of the Araki Neurosurgical Hospital. The studies were conducted in accordance with the local legislation and institutional requirements. Written informed consent for participation was not required from the participants or the participants' legal guardians/next of kin in accordance with the national legislation and institutional requirements.

## Author contributions

ST: Conceptualization, Data curation, Formal analysis, Investigation, Methodology, Supervision, Writing—original draft, Writing—review & editing. TI: Investigation, Methodology, Writing—review & editing. TM: Investigation, Methodology, Writing—review & editing. YI: Investigation, Methodology, Writing—review & editing. RT: Investigation, Methodology, Supervision, Writing—review & editing. NI: Writing—review & editing. HA: Writing—review & editing. OA: Writing—review & editing.
